# Noxious gas detection using carbon nanotubes with Pd nanoparticles

**DOI:** 10.1186/1556-276X-6-605

**Published:** 2011-11-24

**Authors:** Hyang Hee Choi, Junmin Lee, Ki-Young Dong, Byeong-Kwon Ju, Wooyoung Lee

**Affiliations:** 1 Department of Materials Science and Engineering, Yonsei University, Seoul, 120-749, South Korea; 2 Display and Nanosystem Laboratory, College of Engineering, Korea University, Seoul, 136-713, South Korea

## Abstract

Noxious gas sensors were fabricated using carbon nanotubes [CNTs] with palladium nanoparticles [Pd NPs]. An increase in the resistance was observed under ammonia for both CNTs and CNT-Pd sensors. Under carbon monoxide [CO], the two sensors exhibited different behaviors: for CNT sensors, their resistance decreased slightly with CO exposure, whereas CNT-Pd sensors showed an increase in resistance. The sensing properties and effect of Pd NPs were demonstrated, and CNT-Pd sensors with good repeatability and fast responses over a range of concentrations may be used as a simple and effective noxious gas sensor at room temperature.

## Introduction

Carbon nanotubes [CNTs] have a broad variety of structures that have shown applications as materials for a rapid and innovative change in the field of gas sensing [1]. CNTs have recently been proposed as chemical sensors due to their fast response and high sensitivity toward gaseous molecules. However, the chemical and physical interactions between gas molecules and sensing nanotubes are not yet completely understood [2]. Upon exposure to gas molecules, the electrical conductance of CNTs changes and the threshold voltage is shifted due to charge transfer between the semiconducting CNTs and electron-donating (H_2_S, NH_3_, CO)/electron-withdrawing (NO_2_) molecules. Theoretical calculations showed the binding energy of CO and NH_3 _to carbon nanotubes, which indicates a weak charge transfer. The conductivity change may also be caused by contact between the metal electrode and carbon nanotubes and/or the contact between carbon nanotubes [3,4].

CNT-based gas sensors offer significant advantages: unlike oxide-based sensors such as SiO_2 _[5] and ZnO [6] operated at high temperatures for the detection of noxious gases, CNT-based sensors have various merits ranging from a room-temperature operation to a low detection limit. On the other hand, there are several problems to overcome for their practical application. Recently, the combination of CNTs with metal nanoparticles [NPs] has attracted much attention [7-10], given the possibility of use in electronics, as catalysts and as biochemical sensors [11-16]. Some researchers have modified CNTs with Pd NPs using chemical vapor deposition [17], sputtering [18], electron-beam evaporation, thermal evaporation [19,20], dielectrophoresis [21,22], or electrodeposition [23,24]. There have been many efforts to detect noxious gases based on CNTs. In the case of the detection of NH_3_, single-walled carbon nanotube [SWNT]-SnO_2 _sensors can detect a low concentration of 10 ppm NH_3 _gases at room temperature [25]. In addition, in order to improve the sensor's response, some works have been explored with increased operation temperature [26,27]. For the detection of CO, a PANI-functionalized CNT sensor showed a reversible response to CO in the range of 100 to 500 ppm [28], and 10 ppm CO detection at 150°C was reported using WO_3 _films with CNTs [29]. Nevertheless, noxious gas sensing at room temperature using CNT-based sensors appeared to be difficult. In this study, we synthesized noxious gas sensors based on CNTs with reduced Pd NPs. An improvement of the CNTs' response was achieved by employing the reduced Pd NPs, which are likely to react with NH_3 _and CO and result in more stable and sensitive sensors to these gases. The CNT-Pd sensors were highly sensitive to noxious gases with better repeatability and less noise compared with pure CNT sensors, and the differences in sensing properties of the CNTs and CNT-Pd sensors were compared.

### Experimental details

All chemicals were of an analytical reagent grade. Purified arc-discharge nanotubes with a purity of 70% to 90% were purchased from IlJin Nanotech Co., Ltd. (Seoul, South Korea) Sulfuric acid, hydrochloric acid, and nitric acid were purchased from Sigma-Aldrich (St. Louis, MO, USA). Sodium dodecyl sulfate [SDS] surfactant was purchased from Samchun Chemistry (Seoul, South Korea). The purified SWNTs were dissolved in 0.2 wt.% solution of SDS surfactant using deionized water. Dispersion of SWNTs was performed in a bath sonicator for 4 h, and vacuum filtration was performed using Teflon filters (pore size 20 μm, Millipore, Seoul, South Korea). After filtration, the film was rinsed with deionized water for several minutes to remove the SDS surfactant until no bubbles were observed [30].

The reduced CNT-Pd preparation process is described as follows: Both sulfuric and nitric acids (H_2_SO_4_/HNO_3 _= 1:3) were functionalized with carbon nanotubes (1 mg), which were added to an acetone/H_2_O (2:1/*v*:*v*) solution (15 ml), and ultrasonicated for 30 min. For synthesizing the CNT-Pd with an ethylenediaminetetraacetic acid [EDTA]-2Na/Pd ratio of 1:1, the corresponding 0.0186 g of EDTA-2Na in 5 ml of water was added to 10 ml of 5 mM Na_2_PdCl_4_(II) in water. The pH value of the mixture was adjusted to 7 to 8 with an aqueous 0.1 M NaOH solution under vigorous stirring. In the case of the CNT-Pd, 4 ml ethanol was added immediately. The resulting mixture was stirred at 60°C for 3.5 h. Finally, the products were filtered, washed with excess deionized water, and then dispersed in an acetone/H_2_O (2:1 *v*:*v*) solution (15 ml) [31]. A product of reduced Pd on CNTs was fabricated. Transmission electron microscopy [TEM] analysis was performed to investigate the morphology and composition of the reduced CNT-Pd.

**Figure 1:**
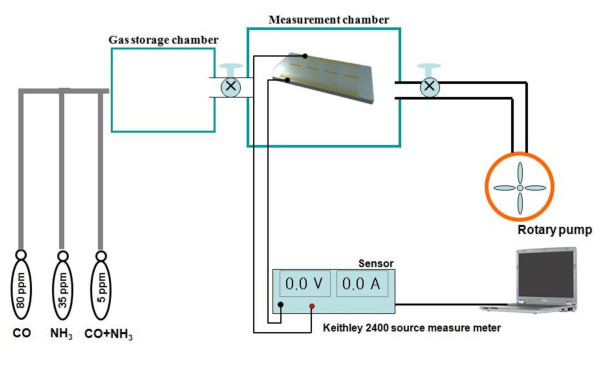
**A schematic of the gas-sensing experimental setup**. (By Choi et al.)

Electrical contacts with the reduced CNT-Pd were created on evaporated 3 nm Cr/50 nm Au electrodes. For gas-sensing experiments, the reduced CNT-Pd was mounted in a small chamber with an electrical feed. The gas-sensing performance of the CNT-Pd sensor was evaluated using a 250-ml test chamber pumped down to 10^-3 ^Torr and filled with a target gas diluted in argon. The test chamber was attached to the control system forming the test bed. Resistance of the CNT-Pd sensor was measured by selecting any two electrodes exhibiting an excellent ohmic contact. The sensor was connected to the gas inlet line from the mass flow controllers. A Keithley model 2400 multimeter (KEITHLEY Instruments, Inc., Cleveland, OH, USA) attached to a computer via a GPIB cable was used to acquire the resistance data using the LabVIEW software (National Instruments, Seoul, South Korea), which was also used to control the mass flow controllers and record the gas concentration, as shown in Figure 1.

## Results and discussion

**Figure 2:**
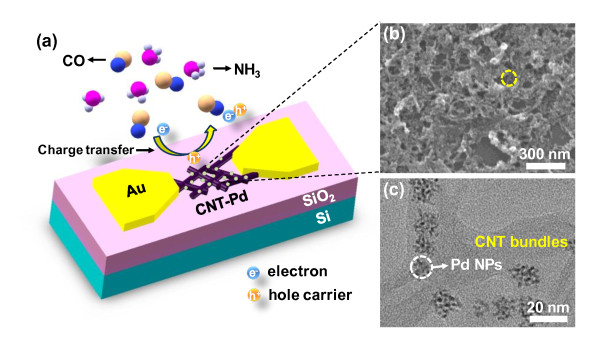
**A gas-sensing device scheme and SEM and TEM images of sensors**. (**a**) A schematic of the random adsorption of gas molecules onto the CNT-Pd sensor. (**b**) An FE-SEM image of the device prepared by dispersing CNTs with Pd NPs. (**c**) An HR-TEM image of CNT bundles with Pd NPs. (By Choi et al.)

A schematic image of the random adsorption of gas molecules on the micro-platform is shown in Figure 2a. CNTs with reduced Pd NPs were dispersed on electrodes prepared using a drop-casting method to fabricate the network-type sensors for detecting CO and NH_3 _at room temperature. When a CNT-Pd sensor is exposed to a noxious gas, the molecules are adsorbed, transferring electrons between the CNT-Pd sensor and the absorbed molecules. Since a Schottky contact is probably formed by Pd NPs on the CNTs, it affects to the hole-carrier mobility of the CNTs. In addition, there is the other possibility of the role of Pd NPs in the sensors: a spill-over effect at the Pd NPs may enhance the sensing abilities of CNT-Pd sensors. This is largely because there are more chances to interact between CNTs and the noxious gases exposed. Consequently, the carrier density and Fermi level of the semiconducting CNT-Pd can be changed. In addition, electrodes may also affect the work function and the electrical properties of the contacted CNTs through modulation of the Schottky barrier [32]. The sensing mechanism related to the charge transfer between gases and sensors is discussed in further detail. Figure 2b shows a field emission scanning electron microscopy [FE-SEM] image of a CNT-Pd sensor, indicating that all parts of the dispersing CNT-Pd were connected in a network-type sensor. Figure 2c shows a high-resolution TEM [HR-TEM] image of CNT-Pd. The average size of Pd NPs on the outer CNT surface was 5 to 10 nm, and no Pd NPs were in contact with each other.

**Figure 3:**
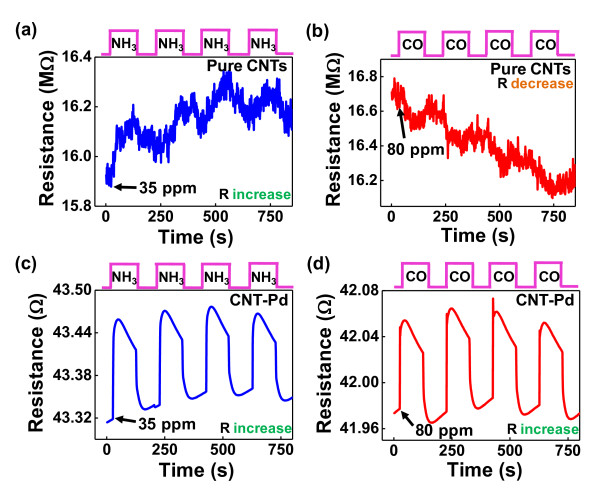
**Plots of real-time electrical resistance responses**. Plots of real-time electrical resistance responses after exposure to 35 ppm NH_3 _and 80 ppm CO for (**a, b**) pure CNT and (**c, d**) CNT-Pd, respectively, at room temperature in air. (By Choi et al.)

Figures 3a, b demonstrate the time dependence of CNT resistance when exposed to 35 ppm NH_3 _and 80 ppm CO, respectively, at room temperature. When electron-donating molecules (NH_3_) interact with the p-type semiconducting CNTs, the electrical resistances of the sensors increase due to the reduced hole carriers in the CNTs. Alternatively, the CO in Figure 3b exhibits different behaviors: the resistance decreased very slightly with exposure of CO in the vacuum, even though previous reports suggest that CO does not react with bare SWNTs [33].

Carboxylic acid group and defect sites may be formed on SWNT sidewalls as a result of purification steps, and interaction with CO molecules likely occurred [34]. Consequently, the COOH functionality and defect sites may play a key role in CO detection, resulting in a decrease in the electrical resistance of CNTs despite the interaction with the electron-donating gas.

For CNTs with Pd NPs, different response properties were observed in Figures 3c, d when exposed to the same gas concentrations. It is inferred that the sensing response was changed by the Pd NPs, such that CNT-Pd sensors increased resistance for the two gases due to the electron transfer to the CNT-Pd sensors. Based on the sensing results, CNT-Pd sensors provided a more reversible reaction, relatively less noise, fast response, good repeatability, a low detection limit, room temperature capability, and complete recovery compared to the abilities of the pure CNT sensors. These responses indicate that the CNT sensors were directly influenced by the properties of the two gases.

**Figure 4:**
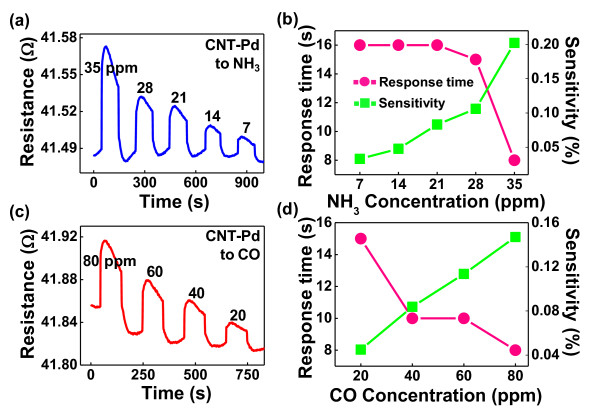
**Plots of real-time electrical resistance responses and their properties for CNT-Pd**. Plots of the real-time electrical resistance responses and their properties (response time and sensitivity) for the CNT-Pd after exposure to (**a, b**) 35 ppm NH_3 _within a concentration range of 7 to 35 ppm and (**c, d**) 80 ppm CO within a concentration range of 20 to 80 ppm. (By Choi et al.)

In Figures 4a, c, the CNT-Pd sensors exhibit fast responses and high sensitivity over a range of concentrations. The sensors detected as low as 7 ppm of NH_3 _and 20 ppm of CO. The sensitivity of the CNT-Pd sensor to noxious gases was defined as:

$$\text{sensitivity}=\frac{R_{\text{after}}-R_{\text{initial}}}{R_{\text{initial}}}\times 100,$$

where *R*_initial _and *R*_after _are the resistances before and after the presence of noxious gases, respectively [35]. Figures 4b, d show that the sensitivity to each gas was nearly linear, so the gas concentrations can be calculated from the sensitivity during noxious gas exposure.

In addition, the response time, defined as the time to reach 90% of the total change in electrical resistance change, was also evaluated [36]. For all gases, CNT-Pd sensors showed response times ranging from 8 to 16 s. The response time was generally faster with increasing gas concentrations. Figures 4b, d can be separated into two parts: the rapid and slow responses. The rapid response arises from molecular adsorption onto the low-energy binding sites, such as *sp*^2^-bonded carbon, and the slow response arises from molecular interactions with higher-energy binding sites, such as vacancies, structural defects, and oxygen functional groups. Adsorption onto an *sp*^2^-bonded carbon occurs through weak dispersive forces; however, at a defect such as a carboxylic acid group, single- and double-hydrogen bonds allow binding energies of at least several hundred millielectron volts/molecule, the main difference between the rapid and slow responses.

## Conclusions

We successfully fabricated a noxious gas sensor for the detection of NH_3 _and CO gases at room temperature using CNTs with reduced Pd NPs. The carboxylic acid group and defect sites appeared to play an important role in the electrical change of pure CNTs to CO. However, the electrical resistance of CNT-Pd sensors increased with exposure to gas via electron transfer. Unlike pure CNT sensors, CNT-Pd sensors exhibited a fast response, linear sensitivity, a low detection limit, and good repeatability over a variety of NH_3 _and CO concentrations and also showed better repeatability and less noise. Moreover, CNT-Pd sensors detected concentrations as low as 7 ppm of NH_3 _and 20 ppm of CO, with a response time of less than 16 s. The characteristics of the CNT-Pd sensors suggest a hopeful candidate for noxious gas sensors, especially those for NH_3 _and CO.

## Abbreviations

CNTs: carbon nanotubes; CO: carbon monoxide; NH_3_: ammonia; Pd: palladium.

## Competing interests

The authors declare that they have no competing interests.

## Authors' contributions

The work presented here was carried out in collaboration among all authors. HHC, JL, and WL defined the research theme. HHC and JL designed the methods and experiments, carried out the laboratory experiments, analyzed the data, interpreted the results, and wrote the paper. K-YD and BK-J worked on the associated data collection and their interpretation and wrote the paper. WL designed the experiments, discussed the analyses, and wrote the paper. All authors have contributed to, seen, and approved the manuscript.
